# Health-related quality of life at 5 years of age for children born very preterm with congenital anomalies: a multi-national cohort study

**DOI:** 10.1038/s41390-024-03521-9

**Published:** 2024-09-07

**Authors:** Sung Wook Kim, Xiaoyu Tian, Lazaros Andronis, Rolf F. Maier, Heili Varendi, Anna-Veera Seppänen, Veronica Siljehav, Elizabeth S. Draper, Jennifer Zeitlin, Stavros Petrou, On behalf of the SHIPS Research Group, J. Lebeer, P. Van Reempts, E. Bruneel, E. Cloet, A. Oostra, E. Ortibus, I. Sarrechia, K. Boerch, P. Pedersen, L. Toome, H. Varendi, M. Männamaa, P. Y. Ancel, A. Burguet, P. H. Jarreau, V. Pierrat, P. Truffert, R. F. Maier, M. Zemlin, B. Misselwitz, L. Wohlers, M. Cuttini, I. Croci, V. Carnielli, G. Ancora, G. Faldella, F. Ferrari, C. Koopman-Esseboom, J. Gadzinowski, J. Mazela, A. Montgomery, T. Pikuła, H. Barros, R. Costa, C. Rodrigues, U. Aden, E. S. Draper, A. Fenton, S. J. Johnson, S. Mader, N. Thiele, J. M. Pfeil, S. Petrou, S. W. Kim, L. Andronis, J. Zeitlin, A. M. Aubert, C. Bonnet, R. El Rafei, A. V. Seppänen

**Affiliations:** 1 Nuffield Department of Primary Care Health Sciences, University of Oxford, Oxford, UK; 2 Kleijnen Systematic Reviews Ltd, York, UK; 3 Warwick Clinical Trials Unit, Warwick Medical School, University of Warwick, Coventry, UK; 4 Children’s Hospital, Philipps University of Marburg, Marburg, Germany; 5 Tartu University Hospital, University of Tartu, Tartu, Estonia; 6 Université Paris Cité, Inserm, INRAE, Centre for Research in Epidemiology and StatisticS (CRESS), Obstetrical Perinatal and Pediatric Epidemiology Research Team, EPOPé, Paris, France; 7 Department of Women’s and Children’s Health, Karolinska Institutet, Stockholm, Sweden; 8 Department of Population Health Sciences, University of Leicester, Leicester, UK; 9 Department of Family Medicine & Population Health (FAMPOP), Disability Studies, Faculty of Medicine & Health Sciences, University of Antwerp, Antwerp, Belgium; 10 Laboratory of Experimental Medicine and Pediatrics, Division of Neonatology, University of Antwerp, Antwerp, Belgium; 11 Study Centre for Perinatal Epidemiology Flanders, Brussels, Belgium; 12 Centre for Developmental Disabilities, Neonatal Intensive Care, Oost Limburg Hospital, Genk, Belgium; 13 Vrije Universiteit Brussel Faculteit Geneeskunde en Farmacie; Paediatric Neurology, Universitair Ziekenhuis Brussels, Jette, Belgium; 14 Centre for Developmental Disabilities, Ghent University Hospital, Gent, Belgium; 15 Centre for Developmental Disabilities, Leuven University Hospital, Leuven, Belgium; 16 Department of Neuropediatrics, University of Leuven, Leuven, Belgium; 17 Department of Paediatrics, Hvidovre Hospital, Copenhagen University Hospital, Hvidovre, Denmark; 18 Department of Neonatology, Hvidovre Hospital, Hvidovre, Denmark; 19 Tallinn Children’s Hospital, Tallinn, Estonia; 20 University of Tartu, Tartu, Estonia; 21 Department of Paediatrics, Institute of Clinical Medicine, University of Tartu, Tartu, Estonia; 22 Division of Pediatrics 2, Hôpital du Bocage; INSERM CIE1, CHRU Dijon, Université de Dijon, Dijon, France; 23 Université Paris Descartes and Assistance Publique Hôpitaux de Paris, Hôpitaux Universitaire Paris Centre Site Cochin, DHU Risks in pregnancy, Service de Médecine et Réanimation Néonatales de Port-Royal, Paris, France; 24 Department of neonatology, Centre Hospitalier Intercommunal Créteil, Créteil, France; 25 Department of Neonatology, Jeanne de Flandre Hospital, Lille CHRU, Lille, France; 26 Children’s Hospital, Philipps University Marburg, University Hospital, Marburg, Germany; 27 University Medical Center, Homburg, Germany; 28 Institute of Quality Assurance Hesse, Eschborn, Germany; 29 Clinical Care and Management Innovation Research Area, Bambino Gesù Children’s Hospital, IRCCS, Rome, Italy; 30 Maternal and Child Health Institute, Marche University and Salesi Hospital, Ancona, Italy; 31 Neonatal Intensive Care Unit, Ospedale degli Infermi, Rimini, Italy; 32 Neonatal Intensive Care Unit, University Hospital S. Orsola-Malpighi, Bologna, Italy; 33 Department of Pediatrics and Neonatology, Modena University Hospital, Modena, Italy; 34 Department of Neonatology, Wilhelmina Children’s Hospital, Utrecht, The Netherlands; 35 Department of Neonatology, Poznan University of Medical Sciences, Poznan, Poland; 36 Department of Neonatology and Neonatal Infectious Diseases, Poznan University of Medical Sciences, Poznan, Poland; 37 EPIUnit-Institute of Public Health, University of Porto, Porto, Portugal; 38 Newcastle University, Newcastle upon Tyne, UK; 39 European Foundation for the Care of Newborn Infants (EFCNI), Munich, Germany; 40 Warwick Medical School, University of Warwick, Coventry, UK

## Abstract

**Background:**

This study aimed to investigate the health-related quality of life (HRQoL) at 5 years of age of European children born very preterm across multi-dimensional outcomes by presence and severity of congenital anomalies.

**Methods:**

The study used data from a European cohort of children born very preterm (<32 weeks of gestation) and followed up to 5 years of age (*N* = 3493). Multilevel Ordinary Least Squares (OLS) regression were used to explore the associations between the presence and severity of congenital anomalies.

**Results:**

The mean total PedsQL™ GCS score for children with a mild congenital anomaly was lower than the respective value for children without a congenital anomaly by 3.7 points (*p* < 0.05), controlling for socioeconomic variables only; this effect was attenuated when accumulatively adjusting for perinatal characteristics (3.3 points (*p* < 0.05)) and neonatal morbidities (3.1 (*p* < 0.05)). The mean total PedsQL™ GCS scores for children who had a severe congenital anomaly were lower by 7.1 points (*p* < 0.001), 6.6 points (*p* < 0.001) and 6.0 points (*p* < 0.001) when accumulatively adjusting for socioeconomic, perinatal and neonatal variables, respectively.

**Conclusion:**

This study revealed that the presence and severity of congenital anomalies are significant predictors of HRQoL outcomes in children born very preterm.

**Impact:**

- Children born very preterm with congenital anomalies experience poorer health-related quality of life (HRQoL) than their very preterm counterparts born without congenital anomalies.
- Increased severity of these anomalies compounds the negative impacts on HRQoL.
- Our findings can be used by stakeholders for clinical and planning purposes.

## Introduction

Congenital anomalies, also known as birth defects or congenital malformations, are structural or functional abnormalities that are present at birth. They represent a significant public health concern, affecting ~3% of US^1^ and UK^2^ newborns, and 2.4% of newborns in Europe.^3^ These conditions contribute to a substantial proportion of infant mortality rates, with ~250,000 deaths attributed to congenital anomalies in 2015 worldwide.^4^

Studies have consistently shown that infants born preterm are more likely to have a congenital anomaly, and infants with a congenital anomaly are more likely to be born preterm. Honein et al.^5^ reported that birth defects were over five times more likely among very preterm births (those at <32 weeks’ gestation) compared with term births in the US using pooled data from 13 states covering the period 1995–2000. In addition, there are a number of studies that quantify the association between congenital anomalies and preterm birth rates in the US,^6,7^ UK,^8^ Denmark,^9^ and Finland.^10^

Despite this association, research is sparse on children born very preterm with congenital anomalies. Children with congenital anomalies are often excluded from research on the causes and neonatal consequences of very preterm birth because of their high mortality and morbidity caused only in part by their prematurity.^11^ Furthermore, in across-country studies, their number and severity can vary in relation to national congenital anomaly screening policies and induced termination of pregnancy policies, which complicate comparisons of outcomes between countries.^12^ Yet, these children and their families face multiple health challenges due to the combination of being born very preterm and having a congenital anomaly; this makes them a vulnerable sub-group that may need targeted intervention and follow-up.

The relationship between gestational age at birth and health-related quality of life (HRQoL) is well-documented with studies generally indicating poorer HRQoL in those born preterm.^13–15^ This holds true when HRQoL is self-reported or reported by parents or proxies.^13,16^ However, some studies suggest that this association does not persist through the lifecourse; for example, a recent systematic review^17^ concluded that there is no definitive evidence for HRQoL differences between term-born and very preterm-born individuals in adulthood.

Nevertheless, existing studies have not investigated the association between the presence and severity of congenital anomalies in very preterm children and the HRQoL of these children. While several studies^18–21^ have explored the relationship between severity of congenital anomalies and HRQoL outcomes, they have not specifically targeted preterm-born children. Additionally, the majority of studies in this area have been conducted in a single country,^10,22–25^ with only a few utilising multinational cohorts.^26^ Some studies have examined the HRQoL of parents caring for children with congenital anomalies,^27–31^ yet these studies did not focus on children born very preterm. By utilising a European preterm birth cohort, we anticipate generating more robust and generalisable findings regarding the HRQoL of children born preterm with congenital anomalies.

Children with congenital anomalies may experience developmental impairment resulting from their specific anomaly independent of their gestational age at birth. This can include physical challenges in, for instance, sitting, walking, or fine motor skills, as well as speech and language delays.^24^ Some congenital anomalies, not only those that primarily affect the brain, can lead to learning disabilities.^25,32,33^ Children might face challenges in school and require special education services. Also, children with visible anomalies or those that affect physical capabilities might face social and emotional challenges, including bullying or social exclusion. These experiences can impact their self-esteem and mental health. It is therefore plausible that impairments in HRQoL are exacerbated in preterm children born with congenital anomalies.

This study had three aims. Firstly, to describe the parent-reported HRQoL of European children born very preterm with congenital anomalies. Secondly, to compare the HRQoL of very preterm children with congenital anomalies with the HRQoL of very preterm children without congenital anomalies. Thirdly, to explore whether the effects on HRQoL vary by severity of congenital anomaly.

## Methods

### Study population

This study utilised data from the European population-based EPICE (Effective Perinatal Intensive care in Europe) cohort of infants born very preterm (<32 weeks’ gestation, *N* = 7900 live births) and the SHIPS (Screening to improve Health In very Preterm infantS) project, which followed-up these children when they were 5 years of age. The cohort comprised all very preterm births over a 12 month period (six months in France) in 2011–2012 in 19 regions across 11 European countries: Belgium (Flanders); Denmark (Eastern Region); Estonia (entire country); France (Burgundy, Ile-de-France and the Northern region); Germany (Hesse and Saarland); Italy (Emilia-Romagna, Lazio and Marche); the Netherlands (Central and Eastern region), Poland (Wielkopolska); Portugal (Lisbon and Northern region); Sweden (Greater Stockholm) and the UK (East Midlands, Northern, and Yorkshire and the Humber regions). Data on pregnancy-related characteristics, perinatal characteristics and neonatal morbidities were gathered from obstetric and neonatal records at birth, and data on HRQoL and sociodemographic characteristics from parental questionnaires completed completed at 5 years of age. More details on the study design, outcome measures, and data collection processes, are available elsewhere.^34^

### Classification of congenital anomalies

Information on congenital anomalies was abstracted from neonatal records and coded from a list of ~70 pre-specified anomalies with the option to record other anomalies using free text. Data were also abstractd on the type of surgery the infant received during the neonatal period for a congenital anomaly as reported by the investigators responsible for data acquisition in study regions. Two senior neonatologists (R.M., H.V.), a paediatric cardiologist (V.S.) and an epidemiologist (E.S.D.) with expertise in congenital anomalies categorised the congenital anomalies listed in the dataset into three categories: mild, moderate, or severe, based on their likely impact on health status. These experts were blinded with respect to the HRQoL outcomes of the study children. Congenital anomalies such as oesophageal atresia or single ventricle physiology of the heart were clearly classified as severe. On the other hand, some anomalies such as isolated hexadactyly anomalies, are known to be mild. However, some of the reported congenital anomalies did not clearly allow a final classification of severity. For example a ventricular septal defect can be either mild or moderate depending on its size, but if diagnosed it most often leads to surgery, which is why it was considered moderate. When a reported malformation was too unspecific, such as “other malformation of the heart”, the child was excluded from further analysis. We restricted our analyses to morphological malformations and excluded inborn errors of metabolism and congenital haematologic or immunologic disorders.

A full description of all the congenital anomalies included in this study, and their designated levels of severity, is reported in Appendix 1.

### Health-related quality of life outcomes

The HRQoL of study children was measured using the Pediatric Quality of Life Inventory (PedsQL™) 4.0 Generic Core Scales (hereafter PedsQL™ GCS for brevity). The PedsQL™ GCS are a collection of validated age-specific questionnaires designed to measure HRQoL in children and adolescents. In this study, parents were asked to complete the PedsQL™ GCS using the parent proxy-report [ages 5–7 (young child)]. The PedsQL™ GCS consists of 23-items covering four sub-scales: physical functioning (8 items), emotional functioning (5 items), social functioning (5 items), and school/daycare functioning (5 items). Each item uses a 5-point Likert scale, with scores ranging from 0 (never a problem) to 4 (almost always a problem). Raw scores are then reversely transformed onto a 0–100 scale, with higher scores indicating better HRQoL. For the computation of sub-scale scores, the sum of item scores is divided by the total number of answered items. This approach accounts for missing data and prevents biased scores when a significant portion of the items are unanswered.^35^ If more than half of the items are missing, the sub-scale score should not be calculated.^35^ A mean psychosocial health summary score was also calculated as the sum of the item scores over the number of the items completed for the emotional, social and school functioning scales, while the physical health summary score was identical to the physical functioning sub-scale score.

### Statistical analysis

The study compared the total PedsQL™ GCS scores of very preterm-born children with congenital anomalies with those without congenital anomalies. The Student’s *t* test was used to test for differences in the total PedsQL™ GCS score and each of the PedsQL™ GCS sub-scale scores. In the same manner, differences in PedsQL™ GCS scores by severity of congenital anomaly (none vs mild, none vs moderate, none vs severe) were also tested using the Student’s t-test.

Ordinary Least Squares (OLS) regression based on multilevel modelling^36^ was used to explore the associations between the congenital anomaly variable of interest and total PedsQL™ GCS score controlling for sociodemographic, perinatal and neonatal variables. The multilevel analysis employed a three-level structure, with individual children at the lowest level, followed by parents (mothers) to account for multiple children per parent, and study country at the highest level in line with published evidence.^14^ Cluster robust standard errors were used to allow for correlations within clusters.^37^

Independent variables used in the modelling were selected based on published evidence,^38–40^ and a published empirical model that used data from the identical cohort.^41^ These included: (i) socioeconomic status (SES) variables—employment status (employed or other situation, at least one parent unemployed), highest maternal education level at 5-year follow-up (high education: International Standard Classification of Education (ISCED) 6–8, intermediate education: ISCED 3-5, low education: ISCED 0-2),^39^ maternal country of birth (native-born, non-native from other European country, non-native from non-European country), and maternal age at childbirth (years) (<25, 25–34, >34); (ii) perinatal variables—parity (primiparous, multiparous), multiplicity (singletons, twins or triplets), sex (male or female), gestational age at birth in completed weeks (<26, 26–27, 28–29, 30–31), and small for gestational age (SGA) status (birth weight for gestational age <3rd percentile, 3rd to 9th percentiles, ≥10th percentile)^42^; and (iii) neonatal morbidities - bronchopulmonary dysplasia (BPD, defined as the need for supplemental oxygen or assisted ventilation at 36 weeks’ postmenstrual age) (no or yes), and severe non-respiratory morbidity (no or yes). The ISCED categorises educational programmes and qualifications by level and field.^43^ ISCED 0 to 2 covers early childhood education through lower secondary education. ISCED 3 to 5 covers upper secondary education through short-cycle tertiary education. Finally, ISCED 6 to 8 covers bachelor’s level education through doctoral education. Severe non-respiratory morbidity (no or yes) was defined as a intraventricular haemorrhage grades III–IV (IVH), cystic periventricular leukomalacia (cPVL), retinopathy of prematurity stages III–V (ROP) or necrotising enterocolitis requiring surgery or peritoneal drainage (NEC).

Three specifications of the multilevel OLS regression models were constructed, informed by evidence from the existing literature^14^: model 1 included (i) SES variables alone; model 2 included (i) SES variables plus (ii) perinatal variables; and model 3 included (i) SES variables, (ii) perinatal variables, and (iii) neonatal morbidities. All three model specifications included congenital anomaly status, (yes, no). The models were also run with congenital anomaly status re-categorised by severity (none, mild, moderate or severe; models 4–6).

A sensitivy analysis was conducted that generated descriptive statistics for the total PedsQL™ GCS score and each of the PedsQL™ GCS sub-scale scores for all categories of the independent variables used in the regression analyses. A further sensitivity analysis excluded children from Denmark and Germany from the multilevel OLS regressions due to the low responses to the PedsQL GCS school functioning questions driven by later compulsory school ages in these countries. All analyses were conducted using Stata 17 (Statacorp, College station, TX).

## Results

**Fig. 1:**
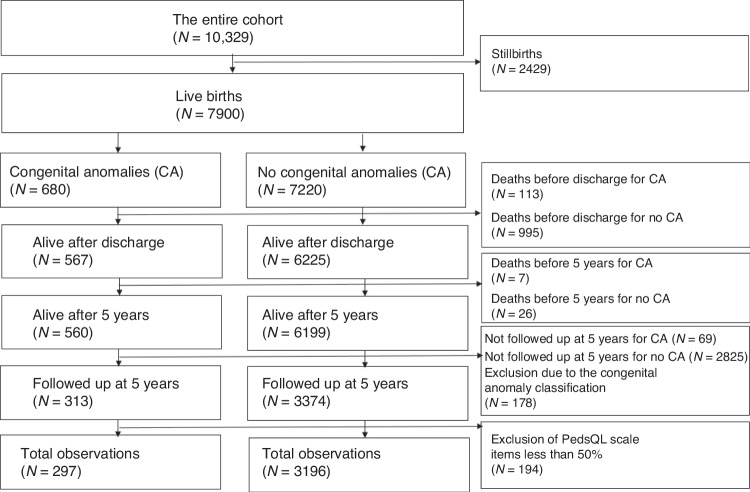
A flow chart of the sample used in this study from the EPICE/SHIPS cohort data.

Tables 1 shows the descriptive characteristics of the study population by congenital anomaly status in the live birth cohort (*N* = 7900) and the follow-up observations used in the analyses in this study (*N* = 3493), respectively (comparative descriptive statistics for each study country are presented in Appendix 2). Of the 7900 live-born children in the cohort, 680 (8.6%) had a congenital anomaly (Table 1), whilst of the 3493 followed up at 5 years of age, 297 (8.5%) had a congenital anomaly (Table 1). A flow chart of the sample used in this study is presented in Fig. 1.

**Table 1:** Descriptive statistics by congenital anomaly status in live births (*N* = 7900). Descriptive statistics by congenital anomaly status in children followed up to 5 years of age (*N* = 3493).

			Congenital anomaly by severity		
	No congenital anomaly	Congenital anomaly	Mild	Moderate	Severe
Total number of observations	7220	680	156	257	267
Gestational age, completed weeks, Mean (SD)	28 (2)	29 (2)	28 (2)	29 (2)	29 (2)
Birth weight (g), Mean (SD)	1194.5 (403.4)	1183.8 (393.6)	1160.9 (361.8)	1164.7 (396.8)	1215.4 (407.4)
Male, *N* (%)	3913 (54.2)	352 (51.8)	73 (46.8)	142 (40.3)	137 (51.3)
Singleton, *N* (%)	4944 (68.5)	493 (72.5)	106 (67.9)	186 (72.4)	201 (75.3)
Birthweight <3rd centile for gestational age, *N* (%)	1431 (19.8)	202 (29.7)	35 (22.4)	84 (32.7)	83 (31.1)
Birthweight 3rd to 9th centile for gestational age, *N* (%)	825 (11.4)	71 (10.4)	22 (14.1)	18 (7.0)	31 (11.6)
Birthweight ≥10th centile for gestational age, *N* (%)	4954 (68.6)	406 (59.7)	99 (63.5)	155 (60.3)	152 (56.9)
Death before discharge	995 (13.8)	113 (16.6)	11 (7.1)	15 (5.8)	87 (32.6)
BPD^a^, *N* (%)	846(11.7)	134 (19.7)	30 (19.2)	58 (22.6)	46 (17.2)
Any severe non-respiratory morbidity^b^, *N* (%)	905 (12.5)	101 (14.9)	24 (15.4)	37 (14.4)	40 (15.0)

	Congenital anomaly		Congenital anomaly by Severity		
	No congenital anomaly	Congenital anomaly	Mild	Moderate	Severe
*N*	3196	297	83	123	91
Gestational age, completed weeks, Mean (SD)	29 (2)	29 (2)	28 (2)	29 (2)	29 (2)
Birth weight (g), Mean (SD)	1243.4 (367.9)	1177.5 (367.6)	1109.4 (346.8)	1169.7 (387.3)	1250.2 (348.8)
Male, *N* (%)	1708 (53.4)	159 (53.5)	37 (44.6)	69 (56.1)	53 (58.2)
Singleton, *N* (%)	2174 (68.0)	205 (69.0)	56 (67.5)	84 (68.3)	65 (71.4)
Birthweight <3rd centile for gestational age, *N* (%)	668 (20.9)	84 (28.3)	18 (21.7)	41 (33.3)	25 (27.5)
Birthweight 3rd to 9th centile for gestational age, *N* (%)	370 (11.6)	35 (11.8)	13 (15.7)	9 (7.3)	13 (14.3)
Birthweight ≥10th centile for gestational age, *N* (%)	2158 (67.5)	178 (59.9)	52 (62.7)	73 (59.3)	53 (58.2)
BPD^c^, *N* (%)	394 (12.3)	55 (18.5)	16 (19.3)	24 (19.5)	15 (16.5)
Any severe non respiratory morbidity^d^, *N* (%)	311 (9.7)	45 (15.2)	19 (22.9)	17 (13.8)	9 (9.9)

^a^*BPD* bronchopulmonary dysplasia. This is defined as receipt of supplemental oxygen and/or ventilatory support (CPAP or mechanical ventilation) at 36 weeks of postmenstrual age.

^b^Any severe nonrespiratory morbidity: IntraVentricular Haemorrhage (IVH) grades III–IV, cystic PeriVentricular Leukomalacia (cPVL), Retinopathy Of Prematurity (ROP) stages III–V or Necrotising EnteroColitis (NEC) needing surgery.

^c^*BPD* bronchopulmonary dysplasia. This is defined as receipt of supplemental oxygen and/or ventilatory support (CPAP or mechanical ventilation) at 36 weeks of postmenstrual age.

^d^Severe non-respiratory morbidity: IntraVentricular Haemorrhage grades III–IV (IVH), cystic PeriVentricular Leukomalacia (cPVL), Retinopathy Of Prematurity stages III–V (ROP) or Necrotising EnteroColitis needing surgery (NEC).

Table 2 presents the mean PedsQL™ GCS scores for children without a congenital anomaly and for children with a congenital anomaly. The table shows the mean scores for each group by PedsQL™ GCS sub-scale, as well as the total PedsQL™ GCS score. The results show that the total PedsQL™ GCS score differed significantly by congenital anomaly status (78.3 for those without a congenital anomaly vs 73.0 for those with a congenital anomaly; *p* < 0. 001). Each sub-scale score also differed significantly by congenital anomaly status: physical functioning (81.0 vs 74.1; *p* < 0.001), emotional functioning (76.0 vs 74.0; *p* = 0.047), social functioning (82.2 vs 76.4; *p* < 0.001), school functioning (76.2 vs 68.3; *p* < 0.001, and psychosocial functioning (78.3 vs 73.0; *p* < 0.001).

**Table 2:** PedsQL™ GCS scores by congenital anomaly status, children followed up to 5 years of age.

	No congenital anomaly	Congenital anomaly	Mean difference	95% Confidence interval	*P* value
	(*N* = 3196^a^), mean (SD)	(*N* = 297), mean (SD)			
Physical functioning	81.0 (20.7)	74.1 (23.5)	6.9	(4.4, 9.4)	<0.001
Emotional functioning	76.0 (17.0)	74.0 (17.5)	2.0	(0.0, 4.1)	0.047
Social functioning	82.2 (18.9)	76.4 (22.0)	5.8	(3.5, 8.1)	<0.001
School functioning	76.2 (19.1)	68.3 (21.3)	7.9	(5.4, 10.4)	<0.001
Psychosocial functioning	79.0 (15.1)	73.3 (16.7)	5.7	(3.9, 7.5)	<0.001
Total score	78.3 (15.1)	73.0 (16.3)	5.3	(3.4, 7.1)	<0.001

^a^Total observations based on the observations used to conduct Student’s *t* test for PedsQL™ GCS scores by congenital anomaly status.

Table 3 presents differences in mean PedsQL™ GCS scores by severity of congenital anomaly. The majority of each of the sub-scale scores were lower in children with a congenital anomaly regardless of the level of its severity. The difference between total PedsQL™ GCS scores by severity of congenital anomaly status were largest for the severe group (8.1; *p* < 0.001). Amongst these children, all the following sub-scale scores were significantly lower than in children without a congenital anomaly: physical functioning (70.7 vs 81.0; *p* < 0.001), social functioning (72.2 vs 82.2; *p* < 0.001), school functioning **(**66.5 vs 76.2; *p* < 0.001, and psychosocial functioning (70.9 vs 78.3; *p* < 0.001). PedsQL™ GCS scale scores by congenital anomaly status are presented in Appendix 3 for all independent variables used in the regression analyses. Within the group of children with congenital anomalies, some high-risk subgroups, such as those with BPD or severe non-respiratory morbidities had lower HRQoL scores compared to children without these conditions. For example, children with both a congenital anomaly and BPD had a mean total score of 66.6, compared to 74.8 for children with a congenital anomaly but without BPD.

**Table 3:** PedsQL™ GCS scores by severity of congenital anomalies

	No congenital anomaly	Mild congenital anomaly	Mean difference	95% Confidence interval	*P* value
	(*N* = 3196), mean (SD)	(*N* = 83), mean (SD)			
Physical functioning	81.0 (20.7)	73.4 (23.0)	7.6	(3.1,12.1)	0.001
Emotional functioning	76.0 (17.0)	71.9 (18.5)	4.1	(0.4,7.9)	0.028
Social functioning	82.2 (18.9)	77.4 (20.1)	4.9	(0.8,9.0)	0.02
School functioning	76.2 (19.1)	66.9 (21.3)	9.3	(4.8,13.8)	<0.001
Psychosocial functioning	78.3 (15.1)	71.9 (16.6)	6.4	(3.1,9.7)	<0.001
Total score	79.0 (15.1)	72.3 (16.8)	6.7	(3.4,10.0)	<0.001
	**No congenital anomaly**	**Moderate congenital anomaly (*N*** = **123), mean (SD)**	**Mean difference**	**95% Confidence interval**	***P*** **value**
	**(*N*** = **3196), mean (SD)**				
Physical functioning	81.0 (20.7)	77.0 (22.0)	4	(0.3,7.7)	0.036
Emotional functioning	76.0 (17.0)	76.1 (16.5)	−0.1	(−3.1,3.0)	0.97
Social functioning	82.2 (18.9)	78.9 (19.6)	3.4	(−0.0,6.8)	0.053
School functioning	76.2 (19.1)	70.2 (20.9)	6	(2.2,9.7)	0.002
Psychosocial functioning	78.3 (15.1)	75.3 (15.2)	3	(0.3,5.7)	0.031
Total score	79.0 (15.1)	75.8 (15.8)	3.2	(0.5,6.0)	0.02
	**No congenital anomaly**	**Severe congenital anomaly**	**Mean difference**	**95% Confidence interval**	***P*** **value**
	**(*N*** = **3196), mean (SD)**	**(*N*** = **91), mean (SD)**			
Physical functioning	81.0 (20.7)	70.7 (25.5)	10.3	(5.9,14.6)	<0.001
Emotional functioning	76.0 (17.0)	73.0 (17.6)	3	(−0.6,6.5)	0.099
Social functioning	82.2 (18.9)	72.3 (25.9)	9.9	(5.9,13.9)	<0.001
School functioning	76.2 (19.1)	66.9 (21.8)	9.4	(4.8,13.9)	<0.001
Psychosocial functioning	78.3 (15.1)	71.0 (17.3)	7.2	(4.1,10.4)	<0.001
Total score	79.0 (15.1)	70.9 (17.6)	8.1	(5.0,11.3)	<0.001

Table 4 shows the results of the multilevel regression analyses based on the presence or absence of a congenital anomaly. The total PedsQL™ GCS score of the children who had a congenital anomaly was lower than the respective value for the children without a congenital anomaly by 4.2 points (*p* < 0.001) in model 1, 3.8 points (*p* < 0.001) in model 2, and 3.4 points (*p* < 0.001) in model 3. In the same manner, male sex (−2.9; *p* < 0.001), being a twin (2.2; *p* < 0.01), at least one parent unemployed (−4.1; *p* < 0.001), maternal education level of low education ISCED 0–2 (−2.2; *p* < 0.05), non-native, non-European maternal birth status (−4.9; p < 0.001) and birth at <26 gestational weeks (−4.8; *p* < 0.001) were statistically significant in model 2. The following variables were also statistically significant in model 3: at least one parent unemployed (−4.2; *p* < 0.001), low level of maternal education (ISCED 0-2) (−2.3; p < 0.01), non-native, non-European maternal birth status (−5.4; *p* < 0.001), being a twin (1.8; *p* < 0.05), male sex (−2.8; *p* < 0.001), presence of BPD (−4.0; *p* < 0.001) and severe non-respiratory morbidity (−5.6; *p* < 0.001).

**Table 4:** Multi-level OLS regression of factors predicting health-related quality of life in very preterm children

		(1)	(2)	(3)		(4)	(5)	(6)
Congenital anomaly (reference: no congenital anomaly)	Yes	−4.2***	−3.8***	−3.4***	Mild	−3.7*	−3.3*	−3.1*
		(0.9)	(0.9)	(0.9)		(1.6)	(1.6)	(1.6)
					Moderate	−2.6*	−2.2	−1.7
						(1.3)	(1.3)	(1.3)
					Severe	−7.1***	−6.6***	−6.0***
						(1.6)	(1.5)	(1.6)
Employment status (reference: Employed or other situation)	At least one parent unemployed	−4.4***	−4.1***	−4.2***	At least one parent unemployed	−4.4***	−4.2***	−4.3***
		(0.9)	(0.9)	(0.9)		(0.9)	(0.9)	(0.9)
Mother’s education (reference: High education ISCED 6–8)	Intermediate education ISCED 3-5	−1.9**	−1.8**	−1.8**	Intermediate education ISCED 3–5	−1.9**	−1.8**	−1.8**
		(0.6)	(0.6)	(0.6)		(0.6)	(0.6)	(0.6)
	Low education ISCED 0-2	−2.3**	−2.2*	−2.3**	Low education ISCED 0-2	−2.2**	−2.1*	−2.3**
		(0.9)	(0.9)	(0.9)		(0.9)	(0.9)	(0.9)
Country of birth for mothers (reference: native)	Non-native, European born	1.7	1.7	2.6	Non-native, European born	1.8	1.7	2.6
		(1.3)	(1.3)	(1.4)		(1.3)	(1.3)	(1.4)
	Non-native, non-European born	−5.1***	−4.9***	−5.4***	Non-native, non-European born	−5.1***	−4.9***	−5.4***
		(0.9)	(0.9)	(0.9)		(0.9)	(0.9)	(0.9)
Mother’s age at childbirth (years) (reference: ^25–34^)	<25	−1.0	−0.8	−0.5	<25	−1.0	−0.8	−0.5
		(0.9)	(0.9)	(0.9)		(0.9)	(0.9)	(0.9)
	>34	1.5*	1.5*	1.4*	>34	1.5*	1.5*	1.4*
		(0.6)	(0.6)	(0.6)		(0.6)	(0.6)	(0.6)
Parity (reference: multiparous)	Nulliparous		0.2	0.2	Nulliparous		0.2	0.2
			(0.6)	(0.6)			(0.6)	(0.6)
Multiples (reference: singleton)	Twins		2.2**	1.8*	Twins		2.2**	1.7*
			(0.7)	(0.7)			(0.7)	(0.7)
	Triplets		−0.2	0.6	Triplets		−0.2	0.6
			(2.2)	(2.2)			(2.2)	(2.2)
Sex (reference: Female)	Male		−2.9***	−2.8***	Male		−2.9***	−2.8***
			(0.5)	(0.5)			(0.5)	(0.5)
SGA (reference: <3rd centile)	3rd to 9th		0.1	−0.2	3–10		0.1	−0.2
			(0.8)	(0.8)			(0.8)	(0.8)
	>10th		0.6	0.1	>10th		0.7	0.1
			(0.6)	(0.6)			(0.6)	(0.6)
Gestational age (weeks) (reference: ^30,31^)	<26		−4.8***	−0.6	<26		−4.8***	−0.6
			(1.0)	(1.1)			(1.0)	(1.1)
	26–27		−1.8*	−0.0	26–27		−1.8*	−0.0
			(0.8)	(0.8)			(0.8)	(0.8)
	28–29		−0.7	−0.3	28–29		−0.7	−0.3
			(0.7)	(0.7)			(0.7)	(0.7)
BPD (reference: no BPD)	Yes			−4.0***	Yes			−4.0***
				(0.8)				(0.8)
Severe non-respiratory morbidity (reference: no severe non-respiratory morbidity)	Yes			−5.6***	Yes			−5.7***
				(0.9)				(0.9)

*SGA* small for gestational age, BPD bronchopulmonary dysplasia. This is defined as receipt of supplemental oxygen and/or ventilatory support (CPAP or mechanical ventilation) at 36 weeks of postmenstrual age.

Severe non-respiratory morbidity: IntraVentricular Haemorrhage grades III–IV (IVH), cystic PeriVentricular Leukomalacia (cPVL), Retinopathy Of Prematurity (ROP) stages III–V or Necrotising CnteroColitis (NEC) needing surgery.

International Standard Classification of Education (ISCED) ISCED 0: Early childhood education (‘less than primary’ for educational attainment) ISCED 1: Primary education ISCED 2: Lower secondary education ISCED 3: Upper secondary education ISCED 4: Post-secondary non-tertiary education ISCED 5: Short-cycle tertiary education ISCED 6: Bachelor’s or equivalent level ISCED 7: Master’s or equivalent level ISCED 8: Doctoral or equivalent level.

Employed or other situation: Other situation included student, parental leave, home parent and other.

Standard errors in parentheses.

***p < 0.001, **p < 0.01, *p < 0.05.

Table 4 also shows the analogous results of the multilevel regression analyses disaggregating by severity of congenital anomaly. The total PedsQL™ GCS score of the children who had a mild congenital anomaly was lower than the respective values for the children without a congenital anomaly by 3.7 points (*p* < 0.05) in model 4, 3.3 points (*p* < 0.05) in model 5 and 3.1 points (*p* < 0.05) in model 6. Likewise, the total PedsQL™ GCS score of the children who had a severe congenital anomaly was lower than the respective values for the children without a congenital anomaly by 7.1 points (*p* < 0.001) in model 4, 6.6 points (*p* < 0.001) in model 5 and 6.0 points (*p* < 0.001) in model 6. Employment status of ‘at least one parent unemployed’, maternal education level of low education ISCED 0-2, non-European maternal birth status, male sex, BPD and severe non-respiratory morbidity continued to have significant negative effects on total PedsQL™ GCS score.

The sensitivity analysis that excluded children from Denmark and Germany revealed similar results to those in Table 4 (Appendix 4). The results of country specific regression analyses are presented in Appendix 5. Appendix 5 demonstrates heterogeneity in HRQoL by congenital status across European countries.

## Discussion

To the best of our knowledge, this is the first study that provides information with respect to HRQoL outcomes at 5 years of age, as measured by the PedsQL™ GCS, in children born very preterm by presence and severity of congenital anomaly across several European countries. The estimated PedsQL™ GCS scores are based on a large sample of 3,493 very preterm born children across Europe.

This study found that the HRQoL of very preterm-born children with a congenital anomaly is significantly poorer than those without a congenital anomaly and that this effect is greater in those with severe congenital anomalies. In all specifications of our multivariate regression models, severe congenital anomalies were associated with an ~6–7 point reduction in the total PedsQL™ GCS score. In the regression models, children with severe congenital anomalies had lower PedsQL™ GCS scores compared to those with mild or moderate congenital anomalies.

In general, the results of this study are consistent with existing research on the impact of congenital heart disease (CHD). Moons et al.^20^ analysed 629 CHD patients in Belgium. They found that scores derived from disease severity classification systems (classification of Task Force 1 of the 32nd Bethesda Conference) were negatively associated with HRQoL and health status and concluded that severity of congenital heart disease is marginally associated with patients’ HRQoL. Knowles et al.^44^ used a UK-wide cohort of children with serious congenital heart diseases aged 10–14 years requiring cardiac intervention during the first year of life in one of 17 UK paediatric cardiac surgical centres operating during 1992–1995. They concluded that children with serious congenital heart diseases experienced poorer HRQoL than unaffected classmates. Mellion et al.^45^ used a US cohort of 1138 children with congenital heart diseases. Children and adolescents with biventricular and single ventricle congenital heart diseases had significantly poorer HRQoL than healthy controls and similar HRQoL as patients with other chronic paediatric diseases. Similar findings have been reported by other congenital heart disease focused studies.^23,45–50^ However, as these studies are limited to the effects of congenital heart disease, direct comparisons with our findings require careful consideration.

This study also found an association between the HRQoL of very preterm born children with congenital anomalies and their parents’ SES. Children with unemployed parents, children with parents with lower levels of education and children of non-European ethnic origins had significantly poorer HRQoL compared to those with employed parent, or with higher education, or of European ethnic origin, according to all multivariate regression models used in this study. The association between the HRQoL of preterm-born children and their parents’ SES has been understudied. Berbis et al.^51^ used a French preterm birth cohort and found that Vécu et Santé Perçue de l’Adolescent et de l’Enfant (VSP-A) scores were associated with family SES; that is, higher SES was significantly positively associated with HRQoL for the RFr (relationships with friends) dimension. Theunissen et al.^16^ investigated the impact of birth factors on HRQoL, rather than SES, using a cohort of 193 children in the Netherlands. They found that SES had a weaker association with HRQoL compared to birth factors, pulmonary disorders and treatments, circulatory disorders, and other health conditions. There are a number of similar studies.^52–55^

Current classification systems for congenital anomalies that have been coded using the ICD-10 classification system do not discriminate by levels of severity. According to the Centers for Disease Control and Prevention, ^56^ major congenital anomalies are conditions that cause most deaths, illnesses, and disabilities related to birth defects, with a list of examples provided, while minor congenital anomalies are conditions that typically do not cause health issues in newborns and have minimal social or cosmetic impact. A study conducted in Denmark^57^ found that Danish mothers giving birth to babies with major congenital anomalies, defined according to the European surveillance of congenital anomalies classification system, have a higher risk of developing new mental health problems. DeSilva et al.^58^ classified congenital anomalies as “major” if they significantly impacted a baby’s lifespan, health, physical abilities, short-term or long-term function whereas “minor” congenital anomalies had little to no effect on these aspects. However, as DeSilva et al.^58^ acknowledged, the study of minor congenital anomalies was beyond the scope of their study. That is to say, due to the relatively weak impact of congenital anomalies classified as “minor” on HRQoL, their definition and research focus remained limited, hindering further investigation. To develop this, we constructed a more detailed categorisation, disaggregating “minor” congenital anomalies into “moderate” and “mild” categories.

One advantage of the classification of congenital anomalies used in our study is that it encompasses all types of congenital anomalies that are experienced by preterm born children, whereas previous studies have focused on a limited number of specific anomalies. For example, Poley et al.^29^ studied HRQoL for children with major congenital anomalies; however, their major congenital anomalies were confined to congenital anorectal malformations or congenital diaphragmatic hernia. In the same manner, Tahirovic et al.^59^ only focused on severity of congenital heart disease. This tendency is observed again in other existing research.^20,49,50^ By moving beyond a focus on specific diseases like congenital heart diseases, our classification system enables the analysis of congenital anomalies for preterm-born children based on level of severity. While further development and validation is required, this classification provides a valuable starting point for analysis in future research on congenital anomalies in the context of preterm birth.

A notable point of this study was that gestational age at birth no longer had a statistically significant effect in our regression models when BPD and severe non-respiratory morbidity were included as independent variables. In general, it is well known that preterm birth is associated with an increased risk of BPD and severe non-respiratory morbidity. These preterm birth-related complications affect HRQoL in preterm born children. However, a recent study of this cohort^14^ showed that the overall HRQoL for very preterm-born children is explained principally by the mediating effects of BPD and severe non-respiratory morbidity rather than by gestational age. In other words, this evidence suggests that addressing the additional long-term impact of preterm birth-related comorbidities such as BPD and brain lesions, and their sequelae, may be important to consider for improving the HRQoL of very preterm-born children with congenital anomalies.

Our regression analyses revealed stronger negative impacts of severe congenital anomalies on the HRQoL of very preterm children compared to mild or moderate congenital anomalies. However, our classification processes acknowledge that some reported congenital anomalies lack clear information for the severity classification. For example, a ventricular septal defect can have a mild or moderate effect on the child depending on if they need surgery or not. We recognise that arbitrariness may exist in our classification of certain congenital anomalies. The classification system was based on the available information at the time, but further refinement is necessary as more data becomes available.

This study has a number of strengths. Firstly, it is based upon a large European sample of 3493 very preterm children followed up to 5 years of age. The recruitment of participants from geographically framed cohorts, rather than from clinical samples, helped to reduce potential selection biases. It was necessary to have a large cohort to generate sufficient numbers of congenital anomalies in the dataset. Despite losing a substantial number of participants to follow-up at 5 years, the descriptive statistics of those followed up remained similar to those of the live birth cohort (Tables 1 and 2). Secondly, the study employed a well-established generic measure of children’s HRQoL, namely the PedsQL™ GCS, which is designed to enable valid comparisons with outcomes in both healthy and clinical populations.^60^ Thirdly, this study encompasses a diverse range of congenital anomalies observed, rather than focusing on a limited selection of congenital anomalies. This comprehensive approach allows for the application of the findings to a broad spectrum of congenital anomalies among very preterm-born children. Lastly, this study was conducted based on a prospective study design that reduce the potential for recall bias, a common bias in retrospective studies.

Some limitations should be acknowledged. Firstly, using parental reports for HRQoL may have introduced reporting biases. However, this is not a unique problem of this study; rather, proxies are recommended for paediatric HRQoL measurement in younger children.^35,61^ There are a number of studies that report children’s HRQoL from both parent and child perspectives in older childhood age groups^60,62^ and future studies need to investigate alternative methods of assessment for younger children to reduce reporting biases caused by proxy reports. Secondly, while a statistically significant difference in PedsQL™ GCS scores was identified between children with and without congenital anomalies, the clinical significance of this difference remains ambiguous. To our knowledge, there are no clear guidelines about what represents a minimum clinically ‘meaningful’ difference in the total PedsQL™ GCS score. Although there are a few studies that have generated estimates of minimally important clinical differences for the PedsQL™ GCS score, these vary by clinical contexts^63–65^ suggesting that they cannot be generalised. Thirdly, due to the extensive amount of loss to follow-up over the first 5 years of life, we conducted analyses on the basis of a smaller sample of 3493 children rather than the 6759 EPICE-SHIPS cohort study children surviving to 5 years. Finally, as discussed above, our classification system for congenital anomalies has limitations with imprecise and income descriptions in some records. Although we excluded cases where severity could not be determined, there still may still be misclassification of cases..

We anticipate that our findings will be a valuable resource for policymaking and further research. By understanding how the presence and severity of congenital anomalies influences the HRQoL of very preterm born children, researchers and decision-makers can better target interventions towards those most affected. Given the impact of congenital anomalies on HRQoL in children born very preterm, specific guidance may be needed within post-discharge follow-up programmes for very preterm-born children on how to improve their physical, emotional, social and school functioning in relation to their congenital anomaly and potentially co-existing developmental delays. All of the 11 countries included in this study currently have national follow-up programmes in place for very preterm and high-risk infants. However, congenital anomalies alone are rarely reported as a criterion for entering these programmes; only six countries follow up these infants until 5 years of age, and only one country (Italy) assesses childrens’ HRQoL as part of this follow-up.^66^ Furthermore, in 2011/2012, when the children in our cohort would have been enroled into these programmes, only three countries (France, Portugal and the Netherlands) provided follow up for children born very preterm until 5 years of age.^66^ Future research should assess whether follow-up programmes targeting very preterm-born children, particularly those with congenital anomalies, could help improve their HRQoL, with particular attention on children from families with social vulnerabilities. Such research, which could include quantitative and qualitative approaches, is needed to better understand what support is required for improving these childrens’ HRQoL, how social circumstances may impede HRQoL, and to offer targed interventions and tailored support for these children.

In terms of the health economic research field, the data presented in this study can be applied in various ways. For instance, our findings could be used to inform the parameterisation of economic models involving very preterm-born children with various congenital anomalies, incorporating the observed differences in HRQoL associated with the severity of congenital anomaly as economic inputs for such models. Ultimately, an optimal level of services or resources allocation to support children with congenital anomalies and their families may be needed to help improve these childrens’ physical, emotional, social and school functioning while improving their HRQoL.

## Conclusion

In conclusion, this study suggests that the HRQoL of very preterm born children is negatively affected by the presence and severity of congenital anomalies. The findings of this study can be used by stakeholders for clinical and planning purposes. Further studies are needed to investigate the optimal allocation of resources for the management of severe congenital anomalies in very preterm born children as these conditions can be highly resource-intensive and potentially impose substantial financial strain on health systems and household resources.

## Data availiability

The data that support the findings of this study were used under license for the current study, and so are not publicly available.

## Supplementary information

Appendix
