# Supplementary material for: Health-related quality of life at 5 years of age for children born very preterm with congenital anomalies: a multi-national cohort study
Source: Pediatr Res. 2024 Sep 7;97(5):1711–21. doi: 10.1038/s41390-024-03521-9 (PMC12119361; doi:10.1038/s41390-024-03521-9)
Supplement: Supplementary file 1 — Appendix [file 41390_2024_3521_MOESM1_ESM.pdf]

Appendix

Appendix 1: Full list of congenital anomalies by severity

Organ System	Anomaly	Classification
Heart	Atrioventricular septal defect (AVSD)	Severe
Heart	Coarctation of the aorta (CoA)	Severe
Heart	Hypoplastic left heart	Severe
Heart	Pulmonary valve atresia	Severe
Heart	Aortic and mitral valves defects	Severe
Heart	Tetralogy of Fallot (ToF)	Severe
Heart	Transposition of great vessels	Severe
Heart	Triscuspidal valve atresia	Severe
Heart	Ventricular septal defect (VSD)	Moderate
Heart	Atrial septal defect (ASD)	Moderate
Heart	Pulmonary valve stenosis	Moderate
Heart	Persistent foramen ovale	Mild

Lung	Pulmonary hypoplasia	Severe
Lung	Cystic adenomatous malformation of lung	Severe
Lung	Chylotorax	Severe
Lung	Diaphragmatic hernia	Severe
Airways	Choanal atresia	Moderate
Airways	Laryngomalacia/Tracheomalacia	Moderate
Airways	Congenital malformation of larynx	Moderate
Airways	Congenital malformation of trachea and bronchus	Moderate
Mouth / Face	Macroglosia	Moderate
Mouth / Face	Cleft lip	Mild
Mouth / Face	Cleft palate	Mild
Mouth / Face	Cleft palate with cleft lip	Mild
Gastrointestinal tract	Oesophageal atresia	Severe

Gastrointestinal tract	Oesophageal atresia with trachea-oesophageal fistula	Severe
Gastrointestinal tract	Duodenal atresia/ileum atresia	Severe
Gastrointestinal tract	Duodenal stenosis/volvulus	Severe
Gastrointestinal tract	Ano-rectal atresia	Severe
Gastrointestinal tract	Hirschsprung disease	Severe
Gastrointestinal tract	Malformation of gallbladder, bile ductus and liver	Severe
Gastrointestinal tract	Gastroschisis	Severe
Gastrointestinal tract	Omphalocele	Severe
Gastrointestinal tract	Ano-rectal stenosis	Moderate
Gastrointestinal tract	Inguinal or umbilical hernia	Mild
Kidney / urinary tract	Cystic kidney disease	Severe
Kidney / urinary tract	Renal agenesis (includes Potter syndrome/fascies)	Severe
Kidney / urinary tract	Renal dysplasia	Severe
Kidney / urinary tract	Ambigous genitalia / indeterminate sex	Severe
Kidney / urinary tract	Hydronephrosis	Moderate
Kidney / urinary tract	Hypospadia	Moderate

Brain / nervous system	Anencephaly	Severe
Brain / nervous system	Microcephaly	Severe
Brain / nervous system	Hydrocephalus	Severe
Brain / nervous system	Myelomeningocele	Severe
Brain / nervous system	Spina bifida	Severe
Brain / nervous system	Malformations of corpus callosum	Mild
Brain / nervous system	Cyst (arachnoid, choroid plexus)	Mild
Skull	Craniosynostosis/scaphocephalia	Severe
Limbs	Sirenomelia	Severe
Limbs	Reduction defects of lower limb	Severe
Limbs	Reduction defects of upper limb	Severe
Limbs	Hip dislocation	Severe
Limbs	Club foot / talipes equinovarus	Moderate
Limbs	Polydactyly	Mild
Limbs	Syndactyly	Mild

Skeletal system	Skeletal Dysplasia	Severe
Skeletal system	Vertebral malformation,	Severe
Skeletal system	Congenital abscence of rib	Mild
Skin	Collodium Baby	Severe
Skin	Hemangioma	Mild
Skin	Aplasia cutis	Mild
Skin	Auricular, preauricular malformation (includes skin tag)	Mild
Eye	Cataract and othe eye malformations	moderate
Chromosomal anomalies	Down's syndrome (trisomy 21)	Severe
Chromosomal anomalies	Edward's syndrome (trisomy 18)	Severe
Chromosomal anomalies	Patau's syndrome (trisomy 13)	Severe
Chromosomal anomalies	Turner's syndrome	Severe

Chromosomal anomalies	Tryploidy	Severe
Complex malformations / Syndroms	Conjoined twins	Severe
Complex malformations / Syndroms	Fetal alcohol syndrome	Severe
Complex malformations / Syndroms	Warfarin syndrome	Severe
Complex malformations / Syndroms	Branchio-oculo facial syndrome	Severe
Complex malformations / Syndroms	CHARGE syndrome	Severe
Complex malformations / Syndroms	Myotonic dystrophia (Steinert)	Severe
Complex malformations / Syndroms	Ellis-van Creveld syndrome	Severe
Complex malformations / Syndroms	Simpson-Golabi-Behmel syndrome	Severe

Complex malformations / Syndroms	Di George syndrome	Severe
Complex malformations / Syndroms	Marfan syndrome	Severe
Complex malformations / Syndroms	Congenital malformation syndromes predominantly associated with short stature (Cornelia de Lange, Prader-willi, Noonan)	Severe
Complex malformations / Syndroms	Congenital malformation syndromes predomiantly affecting facial appearance (Moebius, Schinzel-Giedion)	Severe
Complex malformations / Syndroms	Tuberous sclerosis	Severe
Complex malformations / Syndroms	Sacral teratoma	Severe

#Note:

This list includes those congenital anomalies reported by the treating team in the neonatal period.

Unspecific reported items such as “other anomalies of an organ system” were excluded.

Appendix 2: Descriptive statistics of participant characteristics by country

	Belgium	Denmark	Estonia	France	Germany	Italy	Netherlands	Poland	Portugal	UK	Sweden
**Infants (N)**	752	351	153	1307	758	1134	393	316	724	1745	267
**Gestational age, completed weeks, Mean (SD)**	28.6 (2.3)	28.0 (2.4)	28.7 (2.2)	28.4 (2.3)	28.2 (2.6)	28.5 (2.4)	28.4 (2.5)	28.1 (2.8)	28.5 (2.2)	28.5 (2.4)	28.2 (2.5)
**Male, N (%)**	426 (56.6)	185 (52.7)	80 (52.3)	701 (53.6)	407 (53.7)	563 (49.6)	195 (49.6)	176 (55.7)	423 (58.4)	965 (55.3)	145 (54.3)
**Singleton, N (%)**	464 (61.7)	208 (59.3)	119 (77.8)	881 (67.4)	487 (64.2)	768 (67.7)	274 (69.7)	251 (79.4)	500 (69.1)	1300 (74.6)	185 (69.3)
**Twins or more, N (%)**	288 (38.3)	143 (40.7)	34 (22.2)	426 (32.6)	271 (35.8)	366 (32.3)	119 (30.3)	65 (20.6)	224 (30.9)	443 (25.4)	82 (30.7)
**Birth weight (grams), Mean (SD)**	1260.0 (420.8)	1141.6 (415.4)	1287.0 (390.1)	1162.3 (372.1)	1165.1 (418.0)	1195.4 (420.3)	1198.2 (412.1)	1224.7 (474.2)	1149.4 (363.5)	1210.4 (383.9)	1216.7 (436.4)
**Congenital anomaly, N (%)**	11 (1.5)	23 (6.6)	9 (5.9)	117 (9.0)	99 (13.1)	115 (10.1)	42 (10.7)	90 (28.5)	34 (4.7)	118 (6.8)	22 (8.2)

UK denotes United Kingdom; SD denotes standard deviation.

Appendix 3: PedsQL™ GCS scores by congenital anomaly status and study independent variables

		PedsQL GCS scale	No congenital anomaly, mean (SD)	Congenital anomaly, mean(SD)	Mean difference	95% Confidence interval	P value
**Employment status**	**Employed or other situation**		**N=2839**	**N=254**			
		**Physical functioning**	81.8 (20.2)	74.2 (23.1)	7.6	(5.0, 10.2)	<0.001
		**Emotional functioning**	76.3 (16.7)	74.7 (17.4)	1.6	(-0.5, 3.8)	0.138
		**Social functioning**	82.9 (18.6)	76.4 (21.9)	6.5	(4.1, 8.9)	<0.001
		**School functioning**	77.2 (18.9)	68.9 (21.1)	8.3	(5.7, 11.0)	<0.001
		**Psychosocial functioning**	78.9 (14.9)	73.5 (16.1)	5.4	(3.5, 7.3)	<0.001
		**Total score**	79.7 (14.8)	73.7 (16.5)	6	(4.1, 7.9)	<0.001
**Employment status**	**At least one parent unemployed**		**N=326**	**N=39**			
		**Physical functioning**	74.6 (22.9)	74.8 (25.9)	-0.2	(-8.0, 7.6)	0.959
		**Emotional functioning**	74.2 (18.7)	69.2 (17.6)	5	(-1.2, 11.2)	0.113
		**Social functioning**	77.6 (20.1)	76.6 (23.4)	1.1	(-5.8, 8.0)	0.761
		**School functioning**	69.8 (19.3)	65.4 (23.3)	4.4	(-2.9, 11.7)	0.236
		**Psychosocial functioning**	73.8 (16.1)	69.9 (18.0)	3.9	(-1.5, 9.3)	0.158
		**Total score**	74.1 (16.1)	71.3 (18.4)	2.7	(-2.7, 8.2)	0.325
**Mother's education**	**High education ISCED 6-8**		**N=1326**	**N=104**			
		**Physical functioning**	83.8(19.2)	74.6(22.3)	9.2	(5.3,13.0)	<0.001
		**Emotional functioning**	75.6(16.3)	73.4(16.1)	2.2	(-1.0,5.5)	0.183
		**Social functioning**	83.9(17.6)	73.6(22.4)	10.3	(6.7,13.9)	<0.001
		**School functioning**	79.4(18.1)	69.8(19.5)	9.6	(5.6,13.6)	<0.001

	**Psychosocial functioning**	79.7(14.2)	72.2(15.5)	7.4	(4.6,10.3)	<0.001
	**Total score**	80.8(14.1)	72.9(16.1)	7.9	(5.0,10.7)	<0.001
**Mother's education**	**Intermediate education ISCED 3-5**	**N=1294**	**N=131**			
	**Physical functioning**	80.4(21.4)	74.1(24.2)	6.3	(2.4,10.2)	0.001
	**Emotional functioning**	76.1(17.4)	74.9(17.6)	1.2	(-1.9,4.3)	0.453
	**Social functioning**	82.2(19.0)	78.0(21.4)	4.2	(0.7,7.7)	0.019
	**School functioning**	74.9(19.9)	67.2(22.3)	7.8	(3.9,11.6)	<0.001
	**Psychosocial functioning**	77.8(15.6)	73.5(16.6)	4.3	(1.5,7.1)	0.003
	**Total score**	78.5(15.7)	73.7(17.1)	4.8	(2.0,7.7)	0.001
**Mother's education**	**Low education ISCED 0-2**	**N=495**	**N=57**			
	**Physical functioning**	76.3(20.6)	73.8(24.9)	2.6	(-3.2,8.3)	0.385
	**Emotional functioning**	77.3(17.4)	74.5(17.4)	2.8	(-2.0,7.6)	0.254
	**Social functioning**	79.3(20.7)	78.9(21.9)	0.4	(-5.3,6.1)	0.894
	**School functioning**	71.9(18.3)	68.4(22.5)	3.5	(-2.3,9.4)	0.231
	**Psychosocial functioning**	76.6(15.5)	74.5(15.8)	2.1	(-2.2,6.4)	0.331
	**Total score**	76.6(15.3)	74.2(16.7)	2.3	(-1.9,6.6)	0.278
**Country of birth for mothers**	**Native**	**N=2516**	**N=230**			
	**Physical functioning**	81.8 (20.0)	76.1 (21.9)	5.7	(3.0, 8.5)	<0.001
	**Emotional functioning**	76.0 (16.8)	74.2 (17.2)	1.8	(-0.4, 4.1)	0.112
	**Social functioning**	83.2 (18.2)	77.7 (21.5)	5.5	(3.0, 8.0)	<0.001
	**School functioning**	77.2 (18.7)	70.4 (20.2)	6.8	(4.0, 9.5)	<0.001
	**Psychosocial functioning**	78.9 (14.7)	74.2 (15.9)	4.7	(2.7, 6.7)	<0.001
	**Total score**	79.6 (14.7)	74.7 (16.0)	5	(3.0, 6.9)	<0.001

	**Non native**		**N=502**	**N=46**			
		**Physical functioning**	80.2 (20.6)	75.1 (23.0)	5.1	(1.8, 8.3)	0.002
		**Emotional functioning**	76.4 (17.3)	74.5 (17.0)	1.9	(-0.8, 4.6)	0.164
		**Social functioning**	81.7 (19.2)	78.9 (21.3)	2.8	(-0.2, 5.9)	0.067
		**School functioning**	74.9 (19.2)	68.0 (21.8)	6.9	(3.5, 10.2)	0
		**Psychosocial functioning**	77.9 (15.3)	74.2 (15.8)	3.7	(1.3, 6.1)	0.002
		**Total score**	78.5 (15.1)	74.5 (16.3)	4	(1.7, 6.4)	0.001
**Mother's age at childbirth**	**25-34**		**N=1840**	**N=160**			
		**Physical functioning**	81.1 (20.5)	75.0 (22.6)	6.1	(2.7, 9.4)	<0.001
		**Emotional functioning**	75.5 (16.7)	74.2 (18.2)	1.4	(-1.4, 4.1)	0.325
		**Social functioning**	82.0 (18.7)	75.9 (21.9)	6	(3.0, 9.1)	<0.001
		**School functioning**	76.0 (19.1)	67.9 (20.2)	8.1	(4.7, 11.4)	<0.001
		**Psychosocial functioning**	78.0 (14.9)	72.7 (16.4)	5.3	(2.9, 7.7)	<0.001
		**Total score**	78.8 (14.9)	73.4 (16.5)	5.4	(3.0, 7.9)	<0.001
	**<25**		**N=370**	**N=34**			
		**Physical functioning**	78.5 (23.6)	74.1 (24.2)	4.4	(-3.9, 12.7)	0.299
		**Emotional functioning**	75.9 (17.5)	69.6 (17.8)	6.3	(0.2, 12.5)	0.044
		**Social functioning**	81.2 (19.9)	77.3 (24.0)	3.9	(-3.2, 11.0)	0.284
		**School functioning**	71.6 (19.8)	59.8 (21.6)	11.8	(4.1, 19.6)	0.003
		**Psychosocial functioning**	76.5 (15.7)	69.5 (17.8)	6.9	(1.3, 12.5)	0.016
		**Total score**	77.0 (16.1)	70.5 (17.2)	6.5	(0.8, 12.2)	0.026
	**>34**		**N=986**	**N=103**			
		**Physical functioning**	81.8 (19.8)	72.6 (24.7)	9.1	(5.0, 13.3)	<0.001
		**Emotional functioning**	76.9 (17.2)	75.0 (16.1)	1.8	(-1.6, 5.3)	0.3

		**Social functioning**	83.1 (18.7)	76.9 (21.7)	6.3	(2.4, 10.1)	0.001
		**School functioning**	78.4 (18.5)	71.7 (22.2)	6.7	(2.4, 10.9)	0.002
		**Psychosocial functioning**	79.5 (15.2)	74.6 (15.7)	4.8	(1.7, 7.9)	0.002
		**Total score**	80.1 (15.0)	74.1 (17.0)	6	(2.9, 9.0)	<0.001
**Parity**	**Nulliparous**		**N=1235**	**N=119**			
		**Physical functioning**	79.9 (21.0)	70.8 (25.1)	9.2	(5.1, 13.2)	
		**Emotional functioning**	76.5 (16.9)	74.5 (18.0)	2	(-1.2, 5.2)	
		**Social functioning**	82.4 (19.2)	75.6 (22.8)	6.8	(3.1, 10.5)	
		**School functioning**	75.3 (19.2)	67.5 (21.9)	7.7	(3.8, 11.7)	
		**Psychosocial functioning**	78.2 (15.2)	72.7 (17.0)	5.6	(2.7, 8.5)	
		**Total score**	78.7 (15.2)	72.2 (17.4)	6.5	(3.6, 9.4)	
	**Multiparous**		**N=1931**	**N=169**			
		**Physical functioning**	81.7 (20.4)	76.8 (21.5)	4.9	(1.6, 8.1)	<0.001
		**Emotional functioning**	75.7 (17.0)	73.6 (17.1)	2	(-0.6, 4.7)	0.225
		**Social functioning**	82.2 (18.6)	77.1 (21.5)	5.1	(2.1, 8.1)	<0.001
		**School functioning**	76.9 (19.0)	69.2 (20.7)	7.7	(4.4, 11.1)	<0.001
		**Psychosocial functioning**	78.3 (15.0)	73.4 (15.8)	4.9	(2.5, 7.3)	<0.001
		**Total score**	79.2 (15.0)	74.3 (16.0)	4.9	(2.5, 7.3)	<0.001
**Multiples**	**Singleton**		**N=2174**	**N=205**			
		**Physical functioning**	80.3 (20.8)	73.1 (24.1)	7.2	(4.2, 10.2)	<0.001
		**Emotional functioning**	75.2 (17.1)	73.7 (17.3)	1.5	(-1.0, 3.9)	0.236
		**Social functioning**	81.5 (18.9)	75.3 (21.8)	6.2	(3.5, 9.0)	<0.001
		**School functioning**	75.6 (19.1)	66.7 (21.7)	8.8	(5.8, 11.8)	<0.001
		**Psychosocial functioning**	77.5 (15.2)	72.1 (16.0)	5.4	(3.2, 7.6)	<0.001
		**Total score**	78.3 (15.2)	72.4 (16.7)	5.9	(3.7, 8.1)	<0.001

	**Multiples**		**N=1022**	**N=92**			
		**Physical functioning**	82.4 (20.2)	76.3 (22.1)	6.2	(1.8, 10.5)	0.005
		**Emotional functioning**	77.6 (16.4)	74.4 (17.9)	3.2	(-0.3, 6.7)	0.074
		**Social functioning**	83.9 (18.6)	79.0 (22.3)	4.8	(0.8, 8.9)	0.02
		**School functioning**	77.7 (19.1)	71.8 (19.9)	5.9	(1.3, 10.4)	0.011
		**Psychosocial functioning**	79.9 (14.9)	75.0 (17.0)	4.8	(1.6, 8.0)	0.003
		**Total score**	80.6 (14.8)	75.4 (16.7)	5.2	(2.0, 8.3)	0.002
**Gender**	**Female**		**N=1488**	**N=138**			
		**Physical functioning**	81.5 (20.0)	73.2 (24.4)	8.3	(4.7, 11.8)	<0.001
		**Emotional functioning**	76.6 (16.8)	72.4 (18.7)	4.2	(1.2, 7.2)	0.006
		**Social functioning**	83.7 (17.9)	78.3 (21.1)	5.4	(2.2, 8.5)	0.001
		**School functioning**	79.1 (18.5)	70.6 (21.8)	8.5	(4.9, 12.1)	<0.001
		**Psychosocial functioning**	79.8 (14.7)	73.9 (16.8)	5.9	(3.3, 8.5)	<0.001
		**Total score**	80.3 (14.7)	73.8 (17.1)	6.5	(3.9, 9.1)	<0.001
	**Male**		**N=1708**	**N=159**			
		**Physical functioning**	80.5 (21.2)	74.8 (22.7)	5.7	(2.2, 9.2)	0.001
		**Emotional functioning**	75.4 (17.0)	75.3 (16.2)	0.2	(-2.6, 2.9)	0.899
		**Social functioning**	81.0 (19.5)	74.8 (22.7)	6.2	(3.0, 9.5)	<0.001
		**School functioning**	73.7 (19.3)	66.4 (20.7)	7.3	(3.9, 10.7)	<0.001
		**Psychosocial functioning**	76.9 (15.3)	72.3 (15.9)	4.7	(2.2, 7.2)	<0.001
		**Total score**	77.9 (15.3)	72.9 (16.4)	5	(2.5, 7.5)	<0.001
**SGA**	**<3rd centile**		**N=668**	**N=84**			
		**Physical functioning**	81.3 (19.8)	73.7 (21.6)	7.6	(3.0, 12.1)	0.001
		**Emotional functioning**	75.8 (17.7)	69.3 (18.4)	6.5	(2.5, 10.5)	0.002

		**Social functioning**	81.5 (19.4)	74.1 (21.5)	7.4	(2.9, 11.9)	0.001
		**School functioning**	74.8 (19.4)	64.8 (18.1)	10	(5.3, 14.7)	<0.001
		**Psychosocial functioning**	77.6 (15.5)	69.5 (15.7)	8.1	(4.6, 11.7)	<0.001
		**Total score**	78.6 (15.2)	70.6 (15.8)	8	(4.5, 11.5)	<0.001
	**3rd to 9th**		**N=370**	**N=35**			
		**Physical functioning**	83.1 (18.8)	74.5 (22.7)	8.6	(2.0, 15.3)	0.011
		**Emotional functioning**	75.4 (16.8)	72.4 (16.2)	3	(-2.8, 8.8)	0.31
		**Social functioning**	82.2 (18.3)	78.9 (24.4)	3.3	(-3.3, 9.8)	0.326
		**School functioning**	76.7 (19.0)	70.8 (23.3)	5.8	(-1.4, 13.1)	0.115
		**Psychosocial functioning**	78.1 (14.7)	73.7 (17.6)	4.4	(-0.8, 9.6)	0.098
		**Total score**	79.4 (14.3)	73.9 (18.1)	5.5	(0.4, 10.6)	0.035
	**≥10th centile**		**N=2158**	**N=178**			
		**Physical functioning**	80.5 (21.2)	74.2 (24.6)	6.4	(3.1, 9.6)	<0.001
		**Emotional functioning**	76.2 (16.7)	76.5 (16.8)	-0.3	(-2.9, 2.3)	0.822
		**Social functioning**	82.5 (18.8)	77.0 (21.7)	5.5	(2.5, 8.4)	<0.001
		**School functioning**	76.6 (19.0)	69.6 (22.2)	7	(3.7, 10.3)	<0.001
		**Psychosocial functioning**	78.5 (15.1)	74.6 (16.1)	4	(1.6, 6.3)	0.001
		**Total score**	79.0 (15.2)	74.5 (16.8)	4.6	(2.2, 6.9)	<0.001
**Gestational age**	**<26**		**N=264**	**N=29**			
		**Physical functioning**	76.1 (22.1)	72.3 (21.6)	3.9	(-4.6, 12.3)	0.372
		**Emotional functioning**	73.4 (17.9)	70.3 (20.4)	3	(-4.0, 10.0)	0.394
		**Social functioning**	76.3 (21.1)	73.7 (21.4)	2.6	(-5.5, 10.8)	0.523
		**School functioning**	70.0 (19.1)	64.5 (24.7)	5.5	(-2.6, 13.6)	0.182
		**Psychosocial functioning**	73.5 (16.4)	69.3 (18.5)	4.2	(-2.2, 10.6)	0.196
		**Total score**	74.1 (16.2)	70.0 (18.6)	4.1	(-2.2, 10.5)	0.198

	**26-27**		**N=581**	**N=63**			
		**Physical functioning**	79.9 (22.0)	68.0 (24.3)	12	(6.2, 17.7)	<0.001
		**Emotional functioning**	76.0 (17.3)	72.3 (19.2)	3.7	(-0.9, 8.2)	0.115
		**Social functioning**	81.5 (19.3)	69.6 (20.0)	11.9	(6.8, 17.0)	<0.001
		**School functioning**	75.9 (18.8)	60.9 (20.5)	14.9	(9.5, 20.4)	<0.001
		**Psychosocial functioning**	77.9 (15.4)	67.9 (15.0)	10	(6.0, 14.0)	<0.001
		**Total score**	78.5 (15.7)	68.0 (15.5)	10.5	(6.4, 14.6)	<0.001
	**28-29**		**N=835**	**N=75**			
		**Physical functioning**	80.3 (21.2)	76.1 (22.4)	4.2	(-0.8, 9.3)	0.101
		**Emotional functioning**	76.7 (16.5)	74.7 (18.6)	2	(-1.9, 5.9)	0.321
		**Social functioning**	82.3 (19.2)	79.0 (22.4)	3.4	(-1.2, 8.0)	0.15
		**School functioning**	75.3 (20.5)	71.8 (20.6)	3.5	(-2.0, 8.9)	0.208
		**Psychosocial functioning**	78.3 (15.3)	74.7 (16.8)	3.6	(0.0, 7.3)	0.05
		**Total score**	78.8 (15.3)	75.1 (16.8)	3.8	(0.1, 7.4)	0.043
	**30-31**		**N=1516**	**N=130**			
		**Physical functioning**	82.6 (19.4)	76.3 (23.8)	6.3	(2.8, 9.9)	<0.001
		**Emotional functioning**	76.1 (16.9)	75.1 (15.0)	1	(-2.0, 4.0)	0.53
		**Social functioning**	83.5 (17.9)	78.8 (22.2)	4.7	(1.4, 8.0)	0.005
		**School functioning**	77.9 (18.2)	70.8 (20.4)	7.1	(3.5, 10.7)	<0.001
		**Psychosocial functioning**	79.2 (14.5)	75.4 (15.6)	3.8	(1.2, 6.5)	0.004
		**Total score**	80.1 (14.4)	75.6 (16.3)	4.5	(1.9, 7.1)	0.001
**BPD**	**No**		**N=2726**	**N=234**			
		**Physical functioning**	81.8 (20.1)	76.0 (22.6)	5.8	(3.1, 8.5)	<0.001
		**Emotional functioning**	76.4 (16.6)	74.2 (17.5)	2.2	(-0.1, 4.4)	0.055

		**Social functioning**	83.1 (18.2)	78.5 (22.1)	4.6	(2.1, 7.1)	<0.001
		**School functioning**	77.2 (18.6)	70.1 (20.6)	7	(4.3, 9.8)	<0.001
		**Psychosocial functioning**	79.0 (14.6)	74.3 (16.2)	4.7	(2.7, 6.6)	<0.001
		**Total score**	79.7 (14.6)	74.8 (16.4)	4.9	(3.0, 6.9)	<0.001
**BPD**	**Yes**		**N=394**	**N=55**			
		**Physical functioning**	75.1 (23.8)	66.4 (24.2)	8.7	(2.0, 15.5)	0.011
		**Emotional functioning**	73.4 (19.3)	71.7 (17.4)	1.7	(-3.7, 7.1)	0.534
		**Social functioning**	76.0 (22.3)	67.0 (19.5)	9	(2.8, 15.2)	0.005
		**School functioning**	69.9 (21.2)	61.4 (20.7)	8.6	(2.1, 15.0)	0.01
		**Psychosocial functioning**	73.3 (17.5)	66.8 (14.9)	6.5	(1.6, 11.3)	0.009
		**Total score**	73.7 (17.5)	66.6 (15.7)	7.1	(2.2, 12.0)	0.005
**Severe non-respiratory morbidity**	**No**		**N=2813**	**N=246**			
		**Physical functioning**	82.0 (19.6)	76.4 (21.6)	5.6	(3.0, 8.2)	<0.001
		**Emotional functioning**	76.4 (16.8)	74.7 (17.2)	1.6	(-0.6, 3.8)	0.143
		**Social functioning**	83.1 (18.3)	78.7 (21.9)	4.4	(1.9, 6.8)	<0.001
		**School functioning**	76.9 (18.8)	71.1 (19.4)	5.8	(3.1, 8.5)	<0.001
		**Psychosocial functioning**	78.9 (14.7)	74.8 (15.7)	4.1	(2.1, 6.0)	<0.001
		**Total score**	79.7 (14.6)	75.3 (15.8)	4.5	(2.6, 6.4)	<0.001
**Severe non-respiratory morbidity**	**Yes**		**N=311**	**N=45**			
		**Physical functioning**	72.6 (26.5)	63.4 (28.4)	9.2	(0.8, 17.6)	0.031
		**Emotional functioning**	73.8 (17.4)	69.8 (18.8)	4.1	(-1.5, 9.6)	0.149
		**Social functioning**	75.5 (21.7)	65.6 (19.9)	9.9	(3.1, 16.6)	0.004
		**School functioning**	69.9 (21.1)	56.3 (23.4)	13.7	(6.7, 20.6)	<0.001

**Psychosocial functioning**	73.1 (17.2)	64.0 (16.7)	9.2	(3.8, 14.5)	0.001
**Total score**	73.0 (17.7)	63.9 (18.0)	9.1	(3.5, 14.7)	0.001

SGA: small for gestational age
BPD: bronchopulmonary dysplasia. This is defined as receipt of supplemental oxygen and/or ventilatory support (CPAP or mechanical ventilation) at 36 weeks of postmenstrual age.
Severe non-respiratory morbidity: IntraVentricular Haemorrhage (IVH) grades III-IV, cystic PeriVentricular Leukomalacia (cPVL), Retinopathy Of Prematurity (ROP) stages III-V or Necrotising EnteroColitis (NEC) needing surgery.

International Standard Classification of Education (ISCED) ISCED 0: Early childhood education ('less than primary' for educational attainment) ISCED 1: Primary education ISCED 2: Lower secondary education ISCED 3: Upper secondary education ISCED 4: Post-secondary non-tertiary education ISCED 5: Short-cycle tertiary education ISCED 6: Bachelor's or equivalent level ISCED 7: Master's or equivalent level ISCED 8: Doctoral or equivalent level

Employed or other situation: Other situation included student, parental leave, home parent and other.

Appendix 4: Sensitivity analysis that excludes Denmark and Germany from the multilevel analysis on the total PedsQL GCS score

		(1)	(2)	(3)		(4)	(5)	(6)
**Congenital anomaly (reference: no congenital anomaly)**	**Yes**	-4.1***	-3.5***	-2.9**	**Mild**	-3.5*	-2.8	-2.6
		(0.9)	(0.9)	(0.9)		(1.6)	(1.6)	(1.6)
					**Moderate**	-2.8*	-2.2	-1.6
						(1.4)	(1.4)	(1.4)
					**Severe**	-6.7***	-6.1***	-5.2**
						(1.6)	(1.6)	(1.7)
**Employment status (reference: Employed or other situation)**	**At least one parent unemployed**	-3.9***	-3.6***	-3.9***	**At least one parent unemployed**	-3.9***	-3.7***	-3.9***
		(1.0)	(1.0)	(1.0)		(1.0)	(1.0)	(1.0)
**Mother's education (reference: High education ISCED 6-8)**	**Intermediate education ISCED 3-5**	-2.0**	-1.9**	-1.9**	**Intermediate education ISCED 3-5**	-1.9**	-1.9**	-1.8**
		(0.7)	(0.7)	(0.7)		(0.7)	(0.7)	(0.7)
	**Low education ISCED 0-2**	-2.4**	-2.4*	-2.4*	**Low education ISCED 0-2**	-2.4*	-2.3*	-2.4*
		(0.9)	(1.0)	(1.0)		(0.9)	(1.0)	(1.0)
**Country of birth for mothers (reference: native)**	**Non-native, European born**	1.1	1.1	2.2	**Non-native, European born**	1.1	1.1	2.2
		(1.4)	(1.4)	(1.5)		(1.4)	(1.4)	(1.5)
	**Non-native, non-European born**	-5.4***	-5.2***	-5.7***	**Non-native, non-European born**	-5.4***	-5.2***	-5.8***
		(0.9)	(0.9)	(0.9)		(0.9)	(0.9)	(0.9)
**Mother's age at childbirth (years) (reference: 25-34)**	**<25**	-0.9	-0.7	-0.5	**<25**	-0.9	-0.7	-0.5
		(1.0)	(1.0)	(1.0)		(1.0)	(1.0)	(1.0)

	**>34**	1.8**	1.9**	1.8**	**>34**	1.8**	1.8**	1.7**
		(0.7)	(0.7)	(0.7)		(0.7)	(0.7)	(0.7)
**Parity (reference: multiparous)**	**Nulliparous**		0.3	0.3	**Nulliparous**		0.3	0.3
			(0.6)	(0.6)			(0.6)	(0.6)
**Multiples (reference: singleton)**	**Twins**		2.0**	1.6*	**Twins**		1.9**	1.6*
			(0.8)	(0.8)			(0.8)	(0.8)
	**Triplets**		1.2	2.2	**Triplets**		1.2	2.2
			(2.5)	(2.5)			(2.5)	(2.5)
**Gender (reference: Female)**	**Male**		-3.0***	-2.8***	**Male**		-3.0***	-2.8***
			(0.5)	(0.5)			(0.5)	(0.5)
**SGA (reference: <3rd centile)**	**3rd to 9th**		0.7	0.3	**3 to 10**		0.8	0.4
			(0.9)	(0.9)			(0.9)	(0.9)
	**≥10th**		1.0	0.5	**>10th**		1.0	0.5
			(0.7)	(0.7)			(0.7)	(0.7)
**Gestational age (weeks) (reference: 30-31)**	**<26**		-4.8***	-0.7	**<26**		-4.8***	-0.7
			(1.1)	(1.2)			(1.1)	(1.2)
	**26-27**		-1.7*	0.1	**26-27**		-1.7*	0.1
			(0.8)	(0.9)			(0.8)	(0.9)
	**28-29**		-0.8	-0.4	**28-29**		-0.8	-0.4
			(0.7)	(0.7)			(0.7)	(0.7)
**BPD (reference: no BPD)**	**Yes**			-3.7***	**Yes**			-3.8***
				(0.9)				(0.9)
**Severe non-respiratory morbidity (reference: no severe non-respiratory morbidity)**	**Yes**			-5.9***	**Yes**			-5.9***
				(0.9)				(0.9)

Model 1 and 4 included (i) SES variables alone; model 2 and 5 included (i) SES variables plus (ii) perinatal variables; and model 3 and 6 included (i) SES variables, (ii) perinatal variables, and (iii) neonatal morbidities.

SGA: small for gestational age
BPD: bronchopulmonary dysplasia. This is defined as receipt of supplemental oxygen and/or ventilatory support (CPAP or mechanical ventilation) at 36 weeks of postmenstrual age.
Severe non-respiratory morbidity: IntraVentricular Haemorrhage (IVH) grades III-IV , cystic PeriVentricular Leukomalacia (cPVL), Retinopathy Of Prematurity (ROP) stages III-V or Necrotising CnteroColitis (NEC) needing surgery.
International Standard Classification of Education (ISCED) ISCED 0: Early childhood education (‘less than primary’ for educational attainment) ISCED 1: Primary education ISCED 2: Lower secondary education ISCED 3: Upper secondary education ISCED 4: Post-secondary non-tertiary education ISCED 5: Short-cycle tertiary education ISCED 6: Bachelor’s or equivalent level ISCED 7: Master’s or equivalent level ISCED 8: Doctoral or equivalent level
Employed or other situation: Other situation included student, parental leave, home parent and other.

Standard errors in parentheses

*** p<0.001, ** p<0.01, * p<0.05

Appendix 5: OLS regression of factors predicting health-related quality of life in very preterm children by country

		Belgium	Denmark	Estonia	France	Germany	Italy	Netherlands	Poland	Portugal	UK	Sweden
**Congenital anomaly (reference: no congenital anomaly)**	**Mild**	5.53	-22.18**		2.05	9.27	-6.32*		-5.77		-23.73**	-4.50
		(10.45)	(7.81)		(3.53)	(7.23)	(2.97)		(3.30)		(8.63)	(14.52)
	**Moderate**		3.89	-10.42	-12.79**	-0.59	-2.15	-8.51*	-1.80	4.56	-3.22	-3.34
			(5.67)	(8.53)	(4.45)	(7.30)	(2.73)	(3.46)	(3.91)	(3.98)	(5.15)	(4.46)
	**Severe**		-0.37	-25.84***	-9.30*	-12.49**	-5.23	-5.16	-7.62*	8.03	1.79	-11.62
			(11.10)	(7.39)	(3.79)	(4.61)	(3.70)	(11.90)	(3.75)	(5.54)	(7.57)	(6.14)
**Employment status (reference: Employed or other situation)**	**At least one parent unemployed**	-1.14	-8.94	-16.97***	-3.99*	-13.43*	-0.33	-1.00	-1.86	-0.88	-10.76**	4.00
		(4.74)	(4.79)	(3.92)	(1.81)	(6.54)	(1.65)	(4.82)	(3.31)	(1.79)	(3.78)	(6.54)
**Mother's education (reference: High education ISCED 6-8)**	**Intermediate education ISCED 3-5**	-1.82	0.30	-0.48	-2.71	1.04	-1.45	2.17	2.91	-2.29	-2.52	-5.20*
		(2.27)	(2.24)	(2.31)	(1.46)	(2.71)	(1.17)	(2.24)	(2.38)	(1.52)	(1.93)	(2.43)
	**Low education ISCED 0-2**	-8.30	-4.93	10.48*	-3.90*	-4.80*	-0.61	1.69	-0.60	-4.96**	-2.59	-26.26***
		(5.63)	(4.07)	(4.75)	(1.85)	(2.19)	(1.57)	(4.24)	(4.57)	(1.65)	(5.33)	(6.25)
**Country of birth for mothers (reference: native)**	**Non-native, European born**		-0.78	-47.68***	4.07	9.02*	-0.33	11.55		-7.78*		-14.00**
			(5.17)	(12.50)	(5.35)	(3.92)	(1.51)	(9.63)		(3.64)		(4.83)
	**Non-native, non-European born**	-1.06	4.13		-5.35***	1.44	-5.73**	5.41		-1.21	-5.54	-5.71
		(4.48)	(3.83)		(1.42)	(3.98)	(1.86)	(3.94)		(2.30)	(3.19)	(3.54)

**Mother's age at childbirth (years) (reference: 25-34)**	**<25**	-4.27	0.27	0.66	1.09	4.44	3.63	-8.73*	-0.30	-1.91	2.04	4.69
		(3.44)	(3.70)	(3.33)	(1.92)	(3.69)	(2.31)	(4.21)	(2.87)	(2.12)	(2.78)	(5.13)
	**>34**	-0.14	-2.09	5.56*	0.20	-2.61	2.99**	2.62	-4.75	-1.44	-2.72	0.46
		(2.73)	(2.35)	(2.38)	(1.53)	(2.20)	(1.10)	(2.73)	(2.88)	(1.44)	(2.13)	(2.66)
**Parity (reference: multiparous)**	**Nulliparous**	1.89	1.62	-3.26	-1.85	-4.06	-1.08	5.59*	-0.53	-2.27	1.46	-1.02
		(2.16)	(2.13)	(2.21)	(1.33)	(2.26)	(1.14)	(2.47)	(2.33)	(1.43)	(1.89)	(2.61)
**Multiples (reference: singleton)**	**Twins**	-1.26	6.61**	7.67**	1.22	-2.94	0.65	6.88**	0.04	3.17*	5.95**	-1.72
		(2.13)	(2.22)	(2.97)	(1.36)	(2.19)	(1.18)	(2.43)	(2.70)	(1.40)	(2.12)	(2.51)
	**Triplets**		1.88	9.66*	13.57*	-8.34*	-3.38	6.49	2.82	-0.44	5.93	2.46
			(4.09)	(4.16)	(6.23)	(4.02)	(2.48)	(6.72)	(8.34)	(5.18)	(4.86)	(7.38)
**Gender (reference: Female)**	**Male**	-3.42	-4.12*	-1.94	0.39	-0.08	-1.47	-3.53	-3.00	-3.40**	-4.86**	-1.12
		(2.00)	(2.01)	(2.16)	(1.25)	(1.95)	(1.03)	(2.09)	(2.10)	(1.28)	(1.84)	(2.18)
**SGA (reference: <3rd centile)**	**3rd to 9th**	-0.35	-0.36	-10.74**	0.87	-6.07*	1.50	6.49	-1.16	0.47	1.85	-1.56
		(3.95)	(3.73)	(3.62)	(2.39)	(3.07)	(1.87)	(4.70)	(4.34)	(2.07)	(3.31)	(4.10)
	**≥10th**	-0.60	3.52	-8.00**	0.76	-5.01*	-0.79	2.75	-2.31	0.80	5.15*	2.61
		(2.94)	(2.31)	(2.95)	(1.52)	(2.29)	(1.36)	(2.71)	(3.42)	(1.57)	(2.49)	(3.06)
**Gestational age (weeks) (reference: 30-31)**	**<26**	-2.98	-5.02	12.24*	-0.46	-2.67	2.20	-8.96*	2.27	0.82	-3.20	-10.88**
		(4.12)	(4.08)	(4.84)	(2.96)	(3.40)	(2.40)	(3.75)	(4.00)	(3.42)	(4.05)	(3.64)
	**26-27**	-1.90	-0.72	10.32***	-1.08	-4.89	0.69	-0.54	-5.58	0.74	-1.00	-8.13
		(3.10)	(2.44)	(3.03)	(1.81)	(3.08)	(1.51)	(2.80)	(3.13)	(1.74)	(3.08)	(4.26)
	**28-29**	-2.17	1.77	4.94	0.33	-1.49	1.84	-3.46	-4.11	-0.07	-5.41*	-3.29
		(2.54)	(2.57)	(2.79)	(1.46)	(2.43)	(1.24)	(3.02)	(2.73)	(1.49)	(2.27)	(2.65)
**BPD (reference: no BPD)**	**Yes**	-7.14*	1.93	-10.82**	-1.38	-7.91*	-5.21**	1.04	-1.17	-3.41	-5.10*	4.13
		(3.16)	(3.50)	(3.80)	(2.18)	(3.79)	(2.01)	(2.92)	(4.09)	(2.18)	(2.53)	(3.57)

**Severe non-respiratory morbidity (reference: no severe non-respiratory morbidity)**	**Yes**	-11.67**	1.61	-5.28	-3.28	-2.84	-6.95***	-3.55	-12.18***	-3.98	-1.38	-13.61***
		(3.68)	(3.76)	(3.74)	(2.71)	(3.48)	(1.81)	(3.44)	(2.89)	(2.26)	(3.19)	(4.07)

SGA: small for gestational age
BPD: bronchopulmonary dysplasia. This is defined as receipt of supplemental oxygen and/or ventilatory support (CPAP or mechanical ventilation) at 36 weeks of postmenstrual age.
Severe non-respiratory morbidity: IntraVentricular Haemorrhage (IVH) grades III-IV , cystic PeriVentricular Leukomalacia (cPVL), Retinopathy Of Prematurity (ROP) stages III-V or Necrotising EnteroColitis (NEC) needing surgery.
International Standard Classification of Education (ISCED) ISCED 0: Early childhood education (‘less than primary’ for educational attainment) ISCED 1: Primary education ISCED 2: Lower secondary education ISCED 3: Upper secondary education ISCED 4: Post-secondary non-tertiary education ISCED 5: Short-cycle tertiary education ISCED 6: Bachelor’s or equivalent level ISCED 7: Master’s or equivalent level ISCED 8: Doctoral or equivalent level

Employed or other situation: Other situation included student, parental leave, home parent and other.

Standard errors in parentheses

*** p<0.001, ** p<0.01, * p<0.05
